# Evaluation of *Rz2* Gene Expression in Sugar Beet Hybrids Infected with Beet Necrotic Yellow Vein Virus

**DOI:** 10.3390/cimb46100674

**Published:** 2024-10-12

**Authors:** Ruslan Moisseyev, Alexandr Pozharskiy, Aisha Taskuzhina, Marina Khusnitdinova, Ualikhan Svanbayev, Zagipa Sapakhova, Dilyara Gritsenko

**Affiliations:** 1 Laboratory of Molecular Biology, Institute of Plant Biology and Biotechnology, Almaty 050040, Kazakhstan; rus.mois322@gmail.com (R.M.); aspozharsky@gmail.com (A.P.); ataskuzina@gmail.com (A.T.); germironame@gmail.com (M.K.); uali.svanbayev@gmail.com (U.S.); 2 Department of Molecular Biology and Genetics, Al-Farabi Kazakh National University, Almaty 050040, Kazakhstan; 3 Laboratory of Breeding and Biotechnology, Institute of Plant Biology and Biotechnology, Almaty 050040, Kazakhstan

**Keywords:** *Beta vulgaris* ssp. *vulgaris.* var. *altissima*, BNYVV, rhizomania, resistance, inoculation, RT-qPCR

## Abstract

Sugar beet hybrids are essential in modern agriculture due to their superior yields, disease resistance, and adaptability. This study investigates the role of the *Rz2* gene in conferring resistance to beet necrotic yellow vein virus (BNYVV) in 14 sugar beet hybrids cultivated in Kazakhstan, including local and European varieties. The *Rz2* gene, encoding a CC-NB-LRR protein, is a known resistance factor against BNYVV. Using RT-qPCR, we assessed *Rz2* expression and detected BNYVV in bait plants inoculated with virus-infested soil. Our findings identified two highly resistant varieties: the Kazakh cultivar ‘Abulhair’ and the French line 22b5006. Additionally, the Kazakh cultivar ‘Pamyati Abugalieva’ and the French hybrid ‘Bunker’ exhibited increased resistance, suggesting involvement of other resistance loci. Notably, the Danish hybrid ‘Alando’, despite resistance to rhizomania, did not effectively resist BNYVV, highlighting possible evasion of its genetic factors by local virus strains. Our results emphasize the importance of *Rz2* in resistance breeding programs and advocate for further research on additional resistance genes and the genetic variability of BNYVV in Kazakhstan. This work pioneers the molecular evaluation of BNYVV resistance in sugar beet in Kazakhstan, contributing to sustainable disease management and improved sugar beet production.

## 1. Introduction

Rhizomania caused by beet necrotic yellow vein virus (BNYVV) is one of the most severe diseases affecting sugar beet listed among the quarantine diseases of economic significance [1] and is responsible for sugar beet yield losses of up to 80% [2]. Infected roots exhibit stunted growth, abnormal development of lateral roots around the main root, necrotic rings in the root zone, and yellowing of the leaves [3]. The virus spreads extensively through the soil-borne vector *Polymyxa betae* [4], which produces zoospores in the soil, enabling the virus to survive in the form of resting spores for decades [5]. There are three main pathotypes of the beet necrotic yellow vein virus: A, B, and P [6,7,8]. Pathotype A has been identified in Greece, parts of Europe, the USA, and Asia [8], pathotype B is found in Germany and the Upper Rhine Valley, while pathotype P is limited to specific regions in France, the United Kingdom, and Kazakhstan [6]. Pathotypes A and B contain four genomic RNAs, while pathotype P carries a fifth RNA, which is associated with increased virulence [6,9].

Conventional pest control methods are largely ineffective; thus, the development of resistant sugar beet varieties is of primary importance for crop protection [10]. As of now, four resistance genes, *Rz1*, *Rz2*, *Rz3*, and *Rz4*, have been identified, originating from wild relatives of sugar beet. Genes *Rz1* and *Rz2* are considered the main factors of resistance against BNYVV [11]. Whereas the mechanism of resistance induced by *Rz1* are not known, *Rz2* has been identified as a key component of the plant’s immune defense system, encoding a protein from the CC-NB-LRR class, which plays a role in the active recognition of pathogens [12]. Genes of this type are crucial in providing dominant resistance in plants by initiating a hypersensitive response (HR), leading to localized cell death at the site of infection. R-class proteins are structurally organized into NB and LRR domains, which are responsible for recognizing viral proteins and transmitting signals [13], ultimately triggering cell death, making them essential components of the plant immune response [14]. Experimental studies confirmed that the presence of the *Rz2* gene confers a higher level of resistance [3] by inducing a hypersensitivity response against BNYVV and the beet soilborne mosaic virus belonging to the same genus *Benyvirus* [15]. In 2002–2003, resistance-breaking isolates of BNYVV were detected in the Imperial Valley of California, overcoming the resistance conferred by the *Rz1* gene in sugar beet varieties, whereas *Rz2* retained its effect in prevention of the infection [12].

The present study focuses on the evaluation of resistance against BNYVV conferred by the *Rz2* gene in a selection of local and foreign sugar beet varieties grown in Kazakhstan. The obtained results are novel for Kazakhstan and will help to introduce and implement methods based on molecular biology for the selection of sugar beets resistant to rhizomania and other viral diseases.

## 2. Materials and Methods

### 2.1. Plant Material, Soil Samples, and P. betae Detection

Fourteen sugar beet hybrids from European and Kazakhstani breeding programs were evaluated for resistance to a local isolate of the A strain of BNYVV (Table 1). Six hybrids originated from France, five from Kazakhstan, two from Germany, and one from Denmark. All hybrids had been confirmed to be regionally adapted in the fields of the south and south-east of Kazakhstan. 

Soil samples containing *Polymyxa betae* were collected from sugar beet fields in 2023 and used for plant inoculation with BNYVV. These samples were taken from the collateral root systems of virus-infected plants. The presence of *Polymyxa betae* in the soil was confirmed by PCR using primers PB-F 5′-ATCATGTCGGCAACCGAAAGT-3′ and PB-R 5′-TCTGAGATCTTGTATGGTTCGG-3′, and probe (BHQ)- 5′-TCGGATTCTTGGAACGATAATCCGCCA-3′- (FAM). The primers and probe were developed and provided by LetGen Biotech company (Izmir, Turkey). The reaction mix was prepared using Luna Universal Probe qPCR Master Mix (New England Biolabs, Ipswich, MA, USA) for a volume of 20 μL and contained 0.5 μM each of forward and reverse primers, 0.25 μM probe, and 2 μL of prepared DNA extracts from soil. PCR was carried out using a Bio-Rad CFX 96 system with the following program: initial denaturation at 95 °C for 1 min; 45 cycles of denaturation at 95 °C for 15 s, followed by combined annealing and elongation at 58 °C, with measurements of the signal for 30 s according to manufacturer protocol. The Limit of Detection (LOD) for *P. betae* was 10^1^ copies/µL: Cq = 37.2 ± 0.5. LOD was calculated by standard deviation analysis according to the manufacturer’s protocol. The threshold was set at 95 RFU.

### 2.2. Plant Inoculation and BNYVV Detection

*Polymyxa betae*-positive soil samples were mixed with clean soil in the ratio 1:1. Three to five sugar beet seeds of every hybrid were planted in this soil in three replicates and cultivated in the climatic chamber KBWF 240 (Binder, Tuttlingen, Germany). The temperature and relative humidity were kept at values 24 °C and 65%, respectively. The photoperiod was set to a 14 h light and 10 h dark cycle. The control plants were cultivated in clean soil. The lateral rootlets of the plants were periodically examined for the presence of *P. betae* cystosori by a microscope using 0.05% aniline blue dye in lactoglycerol as a staining and mounting medium.

The total RNA from plants was extracted by an RNA Plant/Fungi Total RNA Purification Kit (Norgen Biotek, Thorold, ON, Canada). For the detection of BNYVV, a Real-Time PCR Detection Kit provided by LetGen Biotech, Turkey (Cat# LSK471-0500), was used. This kit was designed for the direct detection of viral RNA by reverse transcription and qPCR within a single tube and includes two reaction mixes: the first for the detection of pathotype B with an FAM-labeled probe, and the second for the detection of pathotypes A and P with probes labeled with FAM and Cy5, respectively. The reaction mix was prepared following the manufacturer’s recommendation. For each sample, 500 ng of total RNA was used. PCR was run in a Bio-Rad CFX 96 real-time amplification system. A sample was considered positive for BNYVV types A, B, or P if the threshold cycle value was present and did not exceed 30. LOD was 12 copies/µL with a corresponding Ct value of 35.0. LOD was calculated by standard deviation analysis according to the manufacturer’s protocol. The threshold was set at 100 RFU for FAM and 75 for Cy5.

### 2.3. RT-qPCR Analysis of Rz2 Expression

Total RNA was isolated from the lateral root system of each hybrid using the Plant/Fungi Total RNA Purification Kit (Norgen Biotek, Thorold, ON, Canada) according to the manufacturer’s protocol. A total of 1-2 µg of total RNA was used for reverse transcription (RT). RT of obtained RNA extracts was performed for an hour at 45 °C using Superscript IV reverse transcriptase (Thermo Fisher, Waltham, MA, USA), according to the manufacturer’s protocol. A combination of oligo-dT and random hexameric primers was used.

The expression of the gene *Rz2* was analyzed using primers qP-Rz2s (5′- CAGCAGCAATACACAAGTCCA-3′) and qP-Rz2as (5′- TGATGAATGTAATGGAGCATAGAAATT-3′) [15]. The sugar beet *GAPDH* gene was used as a reference gene [15]. The reaction mixes for *Rz2* and reference gene were prepared using Luna Universal qPCR Master Mix (NEB, Ipswich, MA, USA) for a volume of 20 μL and contained 0.4 μM each of forward and reverse primers. PCR was carried out using the Bio-Rad CFX 96 system. The reaction conditions were as follows: an initial denaturation step at 95 °C for 3 min, followed by 40 cycles of denaturation at 95 °C for 30 s, annealing at 60 °C for 20 s, and extension at 72 °C for 40 s. A final extension step was performed at 72 °C for 5 min. Each biological replicate consisted of three different plants, and three biological replicates were used. Each biological sample was analyzed in two technical repeats. Normalization of data and calculation of relative expression values were performed using the 2^−ΔΔCt^ method [16]; the average Ct by biological replicates was used for calculations. The plotting of results was performed using Prism 10.3.1 software for Windows (GraphPad, Boston, MA, USA).

## 3. Results

### 3.1. Analysis of P. betae Infestation and BNYVV Amplification in Lateral Root Systems of Sugar Beet Hybrids

**Figure 1:**
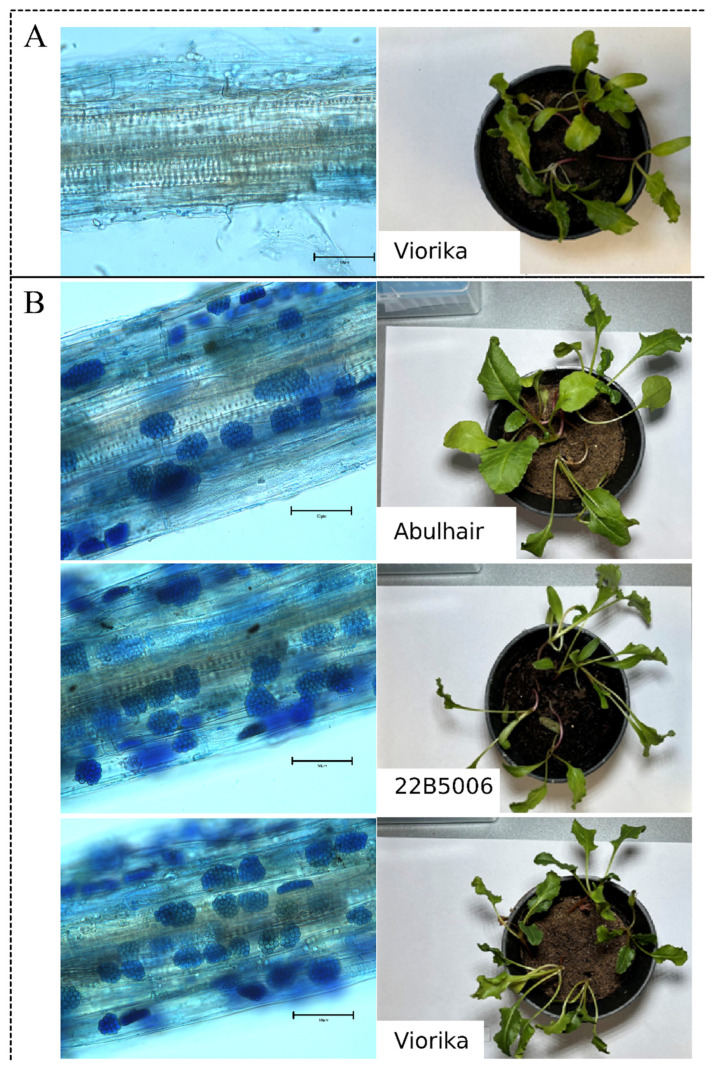
Sugar beet hybrids after 6 weeks of planting in the soil. Microscopy images of stained lateral roots were obtained at 20× magnification (left); scale 50 μm. Infestation by *P. betae* was analyzed in control plants (**A**) grown in sterile soil and in plants grown in *P. betae*-contaminated soil (**B**).

**Figure 2:**
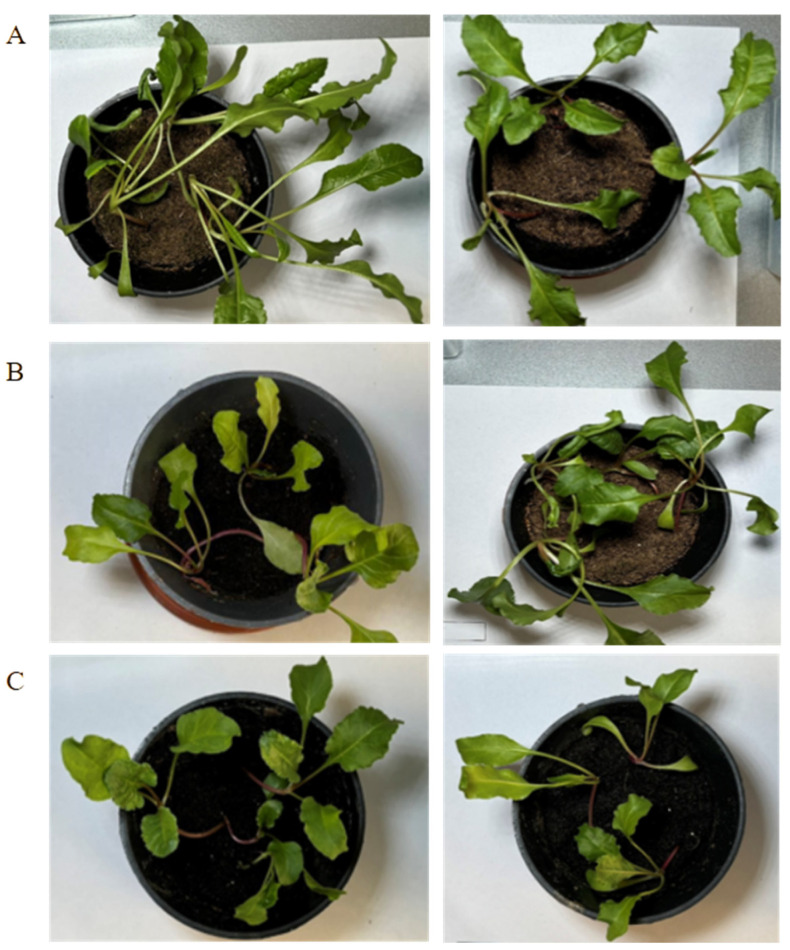
Sugar beet hybrids after 8 weeks of growth in soil. Control healthy plants (left) and BNYVV-infected plants (right). Abulhair (**A**), Viorika (**B**), and Taraz (**C**) hybrids.

To inoculate the sugar beet plants with BNYVV, fourteen hybrids were planted in *P. betae*-contaminated soil samples collected from virus-positive plants in 2023. The isolate Kz1-3 (PP947733.1 and PP947719.1) was used in the present work, which belonged to the A strain according to the qPCR results. The presence of *P. betae* in the soil samples prior to seed planting was confirmed by qPCR. The cultivated plants were screened for the presence of *P. betae* before BNYVV testing. The first signs of *P. betae* infestation in the lateral root systems of the sugar beet plants were observed during the fourth week of cultivation in the contaminated soil. In contrast, the control plants showed no signs of *P. betae* presence. By the fifth and sixth weeks, all experimental plants were infested by *P. betae* (Figure 1). Further, all plants were analyzed by RT-qPCR for virus detection. Despite the presence of BNYVV in the tested plants, symptoms of infection in the above-ground plant parts, such as yellowing, wilting, and leaf chlorosis, were not detected (Figure 2). The evaluation of disease symptoms in the lateral root system could not be conducted due to the plants’ immature stage of development. At this early growth phase, the lateral roots had not sufficiently developed, making it difficult to accurately observe or assess any potential symptoms of disease. 

**Figure 3:**
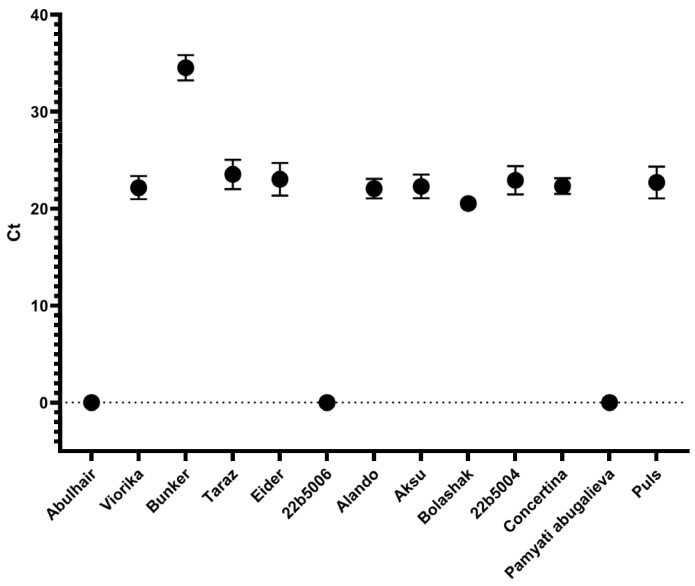
Amplification of BNYVV in 6- and 8-week-old sugar beet hybrids. Ct—cycle threshold. Error bars indicate standard deviation. The paired *t*-test yielded a *p*-value of less than 0.05.

According to the qPCR analysis of BNYVV accumulation in plants, the highest viral amplification was observed in all hybrids at week 6, except for ‘Abulhair’, ‘Pamyati’ Abugalieva, and 22b5006, in which the virus was not detected. For the other hybrids, the Ct values for viral amplification ranged from 20 to 24, indicating a substantial level of viral replication (Figure 3). 

However, for the ‘FD Bunker’ hybrid, the Ct value was notably higher, recorded at 34.5, suggesting a suppression of viral amplification, possibly due to an effective plant defense response. Testing for virus presence at week 8 showed no significant difference in viral amplification compared to week 6, indicating that the viral load stabilized over time in the infected hybrids; Table 2. Therefore, plants from weeks 6 and 8 were used for the analysis of *Rz2* expression. 

### 3.2. Evaluation of Rz2 Expression in Sugar Beet Hybrids

The expression level of the *Rz2* gene was analyzed in all sugar beet hybrids included in the current study. The *Rz2* gene is a key determinant of resistance to BNYVV, and its presence and expression levels can provide insights into the plant’s ability to suppress viral replication. According to the RT-qPCR analysis, the *Rz2* gene was not detected in the ‘Viorika’, ‘Eider’, ‘Alando’, ‘Bolashak’, ‘Concertina’, and ‘Puls’ hybrids. The expression of the *Rz2* gene in the remaining hybrids varied significantly, indicating a differential response to BNYVV infection among the studied sugar beet varieties. The first step was to compare differences in relative expression between weeks 6 and 8. We did not identify statistically supported differences in expression levels (Table 3). The mean values of weeks 6 and 8 were represented further.

**Figure 4:**
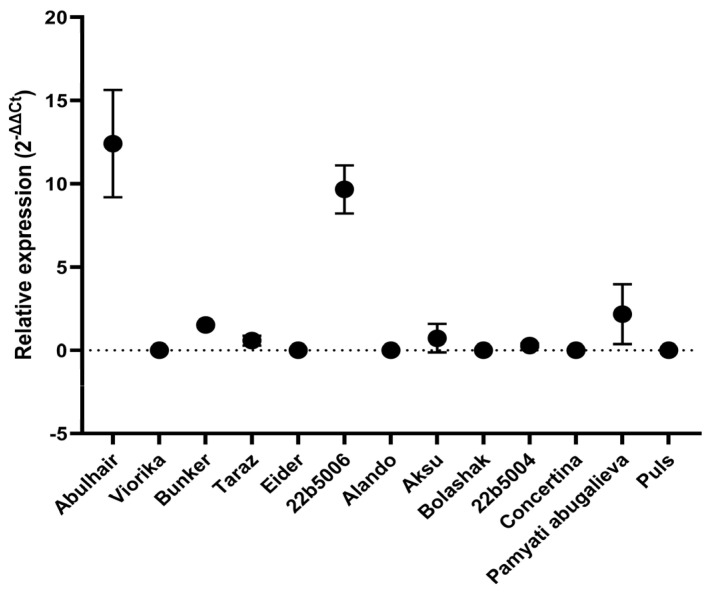
Relative expression of the *Rz2* gene in 6- and 8-week-old sugar beet hybrids was analyzed. The 2^−ΔΔCt^ method was employed to calculate differences in expression between infected and healthy plants, using sugar beet GAPDH as the reference gene. Error bars represent the standard deviation.

The relative expression levels of the *Rz2* gene were highest in the hybrids ‘Abulhair’, ‘22b5006′, and ‘Pamyati Abugalieva’, with mean values of 12.41×, 9.66×, and 2.16×, respectively; Figure 4. These elevated expression levels suggest a strong activation of the plant’s defense mechanisms in these hybrids, potentially contributing to their observed resistance to viral accumulation.

In contrast, the relative expression of *Rz2* in the ‘FD Bunker’, ‘Aksu’, ‘Taraz’, and ‘22b5004′ hybrids was much lower, not exceeding 1.52×. This lower expression may indicate a weaker resistance response, which could be linked to their susceptibility to BNYVV, as observed in the viral amplification analysis. The variability in *Rz2* expression levels across the hybrids highlights the complex nature of resistance mechanisms and suggests that other factors, in addition to *Rz2*, might be influencing the plants’ ability to combat BNYVV infection.

## 4. Discussion

In contemporary agricultural practices, sugar beet hybrids are widely used due to their higher yields, disease resistance, adaptability to diverse climates, and superior root quality [17]. This study aimed to investigate the role of the *Rz2* gene in providing resistance to BNYVV in 14 sugar beet hybrids cultivated in Kazakhstan, including 5 local, 6 French, 2 German, and 1 Danish hybrid. Kazakh hybrids ‘Pamyati Abugalieva’, ‘Aksu’, ‘Abulhair’, ‘Bolashak’, and ‘Taraz’, developed by the Kazakh Research Institute of Agriculture and Plant Growing, are known for their high productivity and disease resistance. The Danish hybrid ‘Alando’ is a high-yielding, mid-season, single-germ diploid with genetic resistance to rhizomania and tolerance to cercospora and aphanomyces, according to the seed provider. The German hybrids ‘Viorika’ and ‘Concertino’ provide substantial sugar production, with ‘Concertino’ excelling in early- and mid-harvest scenarios, while ‘Viorika’ performs well under irrigation and is suitable for mid- to late harvests, with resistance to fusarium, scab, aphanomyces, and cercospora. The French hybrids ‘Bunker’, ‘Eider’, ‘22b5006’, ‘22b5004’, and ‘Puls’, created by PAT “Florimond Desprez Veuve et Fils,” are distinguished by their high sugar content and strong disease resistance. However, for most hybrids the data on their agronomic properties are limited by the commercial information and require experimental validation.

The use of beet hybrids harboring resistance genes against BNYVV is a crucial part of the disease management as the spread of the virus is difficult to control. BNYVV may persist in the dormant spores of *P. betae* for over 15 years [18]; thus, the infested soil may be a source of infection long after the elimination of the affected plants or even crop changes. The use of resistant varieties could increase the efficiency of beet production. The *Rz2* gene encoding the CC-NB-LRR protein is a well-characterized resistance factor against BNYVV in sugar beet [13,19] and considered efficient for disease prevention [20]. Here, we have tested the expression of *Rz2* and the presence of BNYVV in the bait plants after inoculation with soil samples infested by *P. betae* carrying the virus. The tested hybrids represented the sugar beet varieties grown in Kazakhstan, including five hybrids of local selection. The previous studies show that the expression of *Rz2* prevents amplification of the virus by inducing a hypersensitive response-like reaction [15]. As shown in Figure 4, three cultivars demonstrated higher levels of *Rz2* expression. The same three hybrids showed negative results when tested for the presence of BNYVV; Figure 3. Notably, such an outcome was observed regardless of the relative expression levels between these three hybrids. Thus, the expression level observed in the hybrid ‘Pamyati Abugalieva’ was sufficient to prevent virus amplification in plants; however, it could also harbor other resistance loci such as *Rz1*, providing a cumulative resistance effect. Another cultivar, ‘FD Bunker’ (France), had slightly increased expression and showed an increased Ct value, indicating partial suppression of the virus. These hybrids demonstrating suppressed virus with only moderate *Rz2* expression require further extensive studies involving other resistance loci. The obtained results have shown that the expression of the *Rz2* gene measured by RT-qPCR is an informative indicator of resistance in response to the inoculation by BNYVV in sugar beet. As we have seen, the correlation between *Rz2* expression and virus amplification helps to evaluate resistance even when the visual symptoms are absent or week and thus inconclusive.

Based on the obtained results, the hybrid ‘Abulhair’ (Kazakhstan), as well as the line 22b5006 (France), should be considered the promising genetic source for the selection of sugar beets resistant to BNYVV. The hybrid ‘Pamyati Abugalieva’ has also demonstrated efficient prevention of the reproduction of the virus despite lower *Rz2* expression and thus should be tested further. Although allegedly resistant to rhizomania, the ‘Alando’ hybrid did not show efficacy against BNYVV. The reason for this may be that this variety carries genetic factors other than *Rz2*, which could be evaded by the local BNYVV strains.

The present work was the first to evaluate resistance to BNYVV in sugar beet using molecular methods in Kazakhstan. The identified *Rz2* gene should be used as the primary BNYVV resistance factor in domestic breeding programs as it is, unlike the *Rz1* gene, less prone to resistance breaking by genetically diverse BNYVV strains [12,21]. Previously, we found that the strains persisting on local sugar beet fields may be difficult to detect by traditional methods due to a lack of visual symptoms despite causing yield losses [22]. Also, as the previous studies show, the Kazakhstani BNYVV shares peculiar similarity with the P-type isolates from Europe despite the long distance and the limited connections [6]; we have also observed sequence variations in the p25 protein which were not typical for the foreign isolates [22]. Therefore, studies on BNYVV resistance in sugar beet in our country require a comprehensive approach combining expression analysis of resistance genes with wide-scale investigation of the genetic variability in the virus. Thus, the development of new targeted breeding programs is crucial to protect sugar beet production, as well as the development and implementation of sensitive molecular biology-based detection technologies.

Our study focused specifically on the *Rz2* gene in relation to sugar beet resistance, without exploring additional genetic factors that might contribute to enhanced resistance. Further studies ought to focus on additional resistance genes or quantitative trait loci, explore a broader range of varieties, establish several resistance breeding programs, and analyze the long-term resistance stability. Furthermore, gene editing could be investigated to enhance the *Rz2* gene or add novel resistance characteristics into sugar beet. These future directions will enhance the sustainable control of BNYVV and other diseases in sugar beet cultivation, therefore facilitating increased yields, enhanced quality, and the adoption of more sustainable farming methods.

## 5. Conclusions

This study presents the results of tests of the expression of the *Rz2* resistance gene during BNYVV infection in sugar beet varieties grown in Kazakhstan. An RT-qPCR assay in combination with real-time PCR-based BNYVV detection allowed the identification of two highly resistant hybrids: ‘Abulhair’ (Kazakhstan) and the ‘22b5006′ line (France). Additionally, the hybrids ‘Pamyati Abugalieva’ (Kazakhstan) and ‘Bunker’ (France) demonstrated increased resistance in combination with moderate *Rz2* expression, indicating a probable involvement of other resistance loci. Further studies on sugar beet resistance against BNYVV in Kazakhstan require an in-depth investigation of beet resistance with respect to BNYVV’s genetic variability.

## Figures and Tables

**Table 1:** The hybrids of sugar beet of European and Kazakhstani origin.

#	Hybrid	Origin
1	22b5006	France
2	FD Bunker	France
3	Eider	France
4	22b5004	France
5	Puls	France
6	Pamyati Abugalieva	Kazakhstan
7	Aksu	Kazakhstan
8	Abulhair	Kazakhstan
9	Bolashak	Kazakhstan
10	Taraz	Kazakhstan
11	Concertina	Germany
12	Viorika	Germany
13	Alando	Denmark

**Table 2:** RT-PCR analysis of BNYVV amplification in 6- and 8-week-old sugar beet plants. Negative controls were excluded from the table as they were not representative. Paired *t*-test, *p* = 0.907, with *p*-value threshold of 0.05.

			Week 6		Week 8		
Sample ID	Hybrid	*P. betae* in Soil	Ct Value	SD	Ct Value	SD	Presence/Absence of Virus
S1	Abulhair	present	-	-	-	-	Not detected
S2	Viorika	present	22.17	1.20	21.13	0.88	Detected
S3	Bunker	present	34.53	1.30	35.72	1.58	Detected
S4	Taraz	present	23.53	1.50	22.17	1.95	Detected
S5	Eider	present	23.03	1.68	23.57	1.30	Detected
S6	22b5006	present	-	-	-	-	Not detected
S7	Alando	present	22.07	1.00	23.54	1.40	Detected
S8	Aksu	present	22.30	1.22	23.33	1.47	Detected
S9	Bolashak	present	20.53	0.71	19.50	0.84	Detected
S10	22b5004	present	22.93	1.46	21.77	1.46	Detected
S11	Concertina	present	22.33	0.80	21.14	1.32	Detected
S12	Pamyati Abugalieva	present	-	-	-	-	Not detected
S13	Puls	present	22.70	1.64	23.80	1.51	Detected

**Table 3:** RT-qPCR analysis of relative expression of *Rz2* gene in in 6- and 8-week-old sugar beet plants infected by BNYVV. The *Rz2*-negative hybrids were excluded from the table. Paired *t*-test, *p* = 0.9, with *p*-value of threshold 0.05. Also see Table S1 for Ct values for each biological replicate.

		Week 6		Week 8		
Sample ID	Hybrid	ΔΔCt	2^−ΔΔCt^	ΔΔCt	2^−ΔΔCt^	Presence/Absence of Virus
S1	Abulhair	−3.63	12.41	−3.49	11.24	Not detected
S3	Bunker	−0.60	1.52	−0.61	1.53	Detected
S4	Taraz	0.79	0.58	0.91	0.53	Detected
S6	22b5006	−3.27	9.66	−3.01	8.06	Not detected
S8	Aksu	0.47	0.72	0.64	0.64	Detected
S10	22b5004	1.79	0.29	1.58	0.33	Detected
S12	Pamyati Abugalieva	−1.11	2.16	−1.54	2.91	Not detected

## Data Availability

The data generated in this study are included in the article.

## Supplementary Materials

The following supporting information can be downloaded at https://www.mdpi.com/article/10.3390/cimb46100674/s1: Table S1: Ct values for *Rz2* gene expression evaluated by RT-qPCR.

## References

[1] EPPO Datasheet: Beet Necrotic Yellow Vein Virus. https://gd.eppo.int/taxon/BNYVV0/datasheet.

[2] Mcgrann G.R.D., Grimmer M.K., Mutasa-Göttgens E.S., Stevens M. (2009). Progress towards the Understanding and Control of Sugar Beet Rhizomania Disease. Mol. Plant Pathol..

[3] De Biaggi M., Stevanato P., Trebbi D., Saccomani M., Biancardi E. (2010). Sugar Beet Resistance to Rhizomania: State of the Art and Perspectives. Sugar Tech..

[4] Tamada T., Baba T. (1973). Beet Necrotic Yellow Vein Virus from Rizomania-Affected Sugar Beet in Japan. Jpn. J. Phytopathol..

[5] Decroës A., Mahillon M., Genard M., Lienard C., Lima-Mendez G., Gilmer D., Bragard C., Legrève A. (2022). Rhizomania: Hide and Seek of *Polymyxa Betae* and the Beet Necrotic Yellow Vein Virus with Beta Vulgaris. MPMI.

[6] Koenig R., Lennefors B.-L. (2000). Molecular Analyses of European A, B and P Type Sources of Beet Necrotic Yellow Vein Virus and Detection of the Rare P Type in Kazakhstan. Arch. Virol..

[7] Koenig R., Lüddecke P., Haeberlé A.M. (1995). Detection of Beet Necrotic Yellow Vein Virus Strains, Variants and Mixed Infections by Examining Single-Strand Conformation Polymorphisms of Immunocapture RT-PCR Products. J. General. Virol..

[8] Kruse M., Koenig R., Hoffmann A., Kaufmann A., Commandeur U., Solovyev A.G., Savenkov I., Burgermeister W. (1994). Restriction Fragment Length Polymorphism Analysis of Reverse Transcription-PCR Products Reveals the Existence of Two Major Strain Groups of Beet Necrotic Yellow Vein Virus. J. General. Virol..

[9] Koenig R., Haeberlé A.-M., Commandeur U. (1997). Detection and Characterization of a Distinct Type of Beet Necrotic Yellow Vein Virus RNA 5 in a Sugarbeet Growing Area in Europe. Arch. Virol..

[10] Peltier C., Hleibieh K., Thiel H., Klein E., Bragard C., Gilmer D. (2008). Molecular Biology of the Beet Necrotic Yellow Vein Virus. Plant Viruses.

[11] Amiri R., Mesbah M., Moghaddam M., Bihamta M.R., Mohammadi S.A., Norouzi P. (2009). A New RAPD Marker for Beet Necrotic Yellow Vein Virus Resistance Gene in *Beta vulgaris*. Biol. Plant..

[12] Liu H.-Y., Lewellen R.T. (2007). Distribution and Molecular Characterization of Resistance-Breaking Isolates of Beet Necrotic Yellow Vein Virus in the United States. Plant Dis..

[13] Capistrano-Gossmann G.G., Ries D., Holtgräwe D., Minoche A., Kraft T., Frerichmann S.L.M., Rosleff Soerensen T., Dohm J.C., González I., Schilhabel M. (2017). Crop Wild Relative Populations of *Beta Vulgaris* Allow Direct Mapping of Agronomically Important Genes. Nat. Commun..

[14] de Ronde D., Butterbach P., Kormelink R. (2014). Dominant Resistance against Plant Viruses. Front. Plant Sci..

[15] Wetzel V., Willlems G., Darracq A., Galein Y., Liebe S., Varrelmann M. (2021). The *Beta Vulgaris*-Derived Resistance Gene *Rz2* Confers Broad-Spectrum Resistance against Soilborne Sugar Beet-Infecting Viruses from Different Families by Recognizing Triple Gene Block Protein 1. Mol. Plant Pathol..

[16] Livak K.J., Schmittgen T.D. (2001). Analysis of Relative Gene Expression Data Using Real-Time Quantitative PCR and the 2^−ΔΔC_T_^ Method. Methods.

[17] Richardson K. (2010). Traditional Breeding in Sugar Beet. Sugar Tech..

[18] Scholten O.E., Lange W. (2000). Breeding for Resistance to Rhizomania in Sugar Beet: A Review. Euphytica.

[19] Funk A., Galewski P., McGrath J.M. (2018). Nucleotide-Binding Resistance Gene Signatures in Sugar Beet, Insights from a New Reference Genome. Plant J..

[20] Nalbandyan A.A., Fedulova T.P., Hussein A.S. (2019). Molecular Selection of *Beta Vulgaris*, L. Breeding Material with Biotic Stress-Resistance Genes. Russ. Agric. Sci..

[21] Liebe S., Wibberg D., Maiss E., Varrelmann M. (2020). Application of a Reverse Genetic System for Beet Necrotic Yellow Vein Virus to Study *Rz1* Resistance Response in Sugar Beet. Front. Plant Sci..

[22] Pozharskiy A., Mendybayeva A., Moisseyev A., Khusnitdinova M., Nizamdinova G., Gritsenko D. (2024). Molecular Detection and Sequencing of Beet Necrotic Yellow Vein Virus and Beet Cryptic Virus 2 in Sugar Beet from Kazakhstan. Front. Microbiol..

